# Prognostic factors associated with overall survival in patients with oral cavity squamous cell carcinoma

**DOI:** 10.4317/medoral.23558

**Published:** 2020-06-10

**Authors:** Letícia L. Oliveira, Anke Bergmann, Andréia C. Melo, Luiz C. S. Thuler

**Affiliations:** 1 Clinical Research Division, Instituto Nacional de Câncer (INCA), Rio de Janeiro, Brasil

## Abstract

**Background:**

Low socioeconomic status, increasing age, and poor lifestyle behaviors are associated with poor survival in patients with oral cavity squamous cell carcinoma (OCSCC). To determine the overall survival (OS) and the risk of OCSCC death by tumor subsite.

**Material and Methods:**

A retrospective cohort study of OCSCC patients diagnosed from 2007 to 2009 and treated at a single cancer center in Rio de Janeiro, Brazil. Patient information was obtained from the Hospital Cancer Registry (HCR) database and complemented by individual search of physical and electronic medical records. Descriptive statistics of population characteristics were computed. OS was estimated using the Kaplan-Meier method. Univariate and multivariate Cox proportional hazards regression analyses were used to estimate the risk of death by tumor subsite.

**Results:**

Seven hundred and three patients with OCSCC were identified. Most patients were men (77.4%) with low levels of education (67.5%), who drank (73.9%) and smoked (79.7%). The most prevalent tumor site was the tongue (45.4%), 73.4% of patients had advanced (clinical stage III or IV) OCSCC at diagnosis and 74.1% died during follow-up. For the entire cohort, the OS was 39.1% at two years and 27.9% at five years. The median survival time was 1.4 years (95%CI: 1.2‒1.5). Non-operative treatment (HR: 3.11; 95%CI: 2.26‒4.29; *p* <0.001), advanced stage (HR 2.14; 95%CI 1.68-2.74; *p* <0.001), and age >60 years at diagnosis (HR: 1.37; 95%CI: 1.15‒1.64; *p* <0.001) were independently associated with the risk of death. However, these factors varied by tumour subsite.

**Conclusions:**

Analysis of specific subsites of the oral cavity revealed substantial differences in prognostic factors associated with poor survival in OCSCC.

** Key words:**Squamous cell carcinoma, oral cavity cancer, survival, prognosis.

## Introduction

Oral cavity cancer (OCC) is among the most common types of cancer worldwide. In 2018, there were an estimated 354,864 new cases of OCC worldwide. Age-adjusted incidence rates were estimated at 5.8 per 100,000 men and 2.3 per 100,000 women, whereas adjusted mortality rates were estimated at 2.8 per 100,000 in men and 1.2 per 100,000 in women (1). In Brazil, oral cancer is the 7th most incident cancer in the general population, corresponding to a risk of 14.14 new cases per 100,000 (2). Between 2002 and 2013, the adjusted mortality coefficient presented stability in both men and women with an average rate of 1.87 per 100,000 inhabitants (3).

Squamous cell carcinoma (SCC) is the most common histologic type of OCC. As with most cancers, the stage at diagnosis of OCC is highly correlated with the likelihood of survival. However, only 31% of OCC cases are diagnosed at an early stage, when five-year overall survival rates may reach 80% (4,5). However, when diagnosed at a late stage, five-year overall survival ranges from 30% to 50% (5).

In patients with oral cavity squamous cell carcinoma (OCSCC), the main prognostic factors associated with poor survival are age ≥ 60 years, male gender, race/skin color other than white, advanced stage disease at diagnosis, non-eligibility for surgery, and local recurrence (6-9). However, prognostic factors may vary in importance by tumor subsite. To the best of our knowledge, no studies so far have examined the prognostic factors of OCSCC located in distinct subsites of the oral cavity in Brazil. Thus, this study aimed to identify the prognostic factors associated with poor survival by tumor subsite in Brazilian patients diagnosed with OCSCC.

## Material and Methods

This is a retrospective cohort study of patients with OCSCC diagnosed and treated at the Cancer Hospital I of the Brazilian National Cancer Institute (HCI/INCA), Rio de Janeiro, Brazil between January 1, 2007 and December 31, 2009. Tumor subsites were defined according to the International Classification of Diseases for Oncology, third edition (ICD-O-3) as follows: tongue (C02, C02.0, C02.1, C02.2, C02.3, C02.4, C02.8, C02.9); gum (C03, C03.0, C03.1, C03.9); floor of mouth (C04, C04.0, C04.1, C04.8, C04.9); palate (C05, C05.0, C05.1, C05.2, C05.8, C05.9); and other and unspecified parts of mouth (C06, C06.0, C06.1, C06.2, C06.8, C06.9). Patients with tumors of the lip (C00, C00.0, C00.1, C00.2, C00.3, C00.4, C00.5, C00.6, C00.8, C00.9) were not included because of their different risk factors and prognosis. Patients under 18 years of age and patients with synchronous tumors were excluded from the analysis.

Patients were selected from the HCI/INCA Hospital Cancer Registry (HCR). All cases registered between 2007 and 2009 were examined to ensure that patients were followed for at least five years for the calculation of survival probabilities.

Patient information was obtained from the HCR database and complemented by individual search of physical and electronic medical records when needed. Despite efforts, a proportion of patients remain without valid information for some important variables, due to under-registration, errors in the coding of data, misclassification or inconsistencies. The Brazilian Mortality Information System was consulted for all patients lost to follow-up.

The five-year overall survival (OS) rate was the primary outcome (dependent variable), defined as the time interval in years between the date of diagnosis and the date of death from any cause. Patients lost to follow-up were censored at the time of the last HCI/INCA visit.

The independent variables and descriptors examined were gender, age, alcohol use (never drinker, current drinker, former drinker), smoking (never smoker, current smoker, former smoker), race/skin color (classified as white, black, yellow, brown, indigenous according to the Brazilian Institute of Geography and Statistics [IBGE]), clinical stage, tumor size, number of positive lymph nodes, education, tumor-free resection margins, extracapsular spread, grade, tumor topography and morphology, and treatment.

- Statistical analysis

Continuous variables are presented as mean ± standard deviation (SD) whereas categorical variables are presented as frequency distribution. Overall survival was estimated using the Kaplan-Meier method and differences between exposure groups were estimated by using the log-rank test. Univariate and multivariate Cox proportional hazards regression analyses were used to estimate the risk of death by tumor subsite. Variables with a significance level of P < 0.20 on the univariate analysis were included in the multivariate models by stepwise forward selection with the entry order based on their level of significance. Variables of clinical and epidemiological importance were included in the model irrespective of the level of significance on the univariate analysis. The data were entered into an Excel spreadsheet (Microsoft, Redmond, WA, USA) and all analyses were performed using SPSS Statistics for Windows, version 23.0 software (IBM Corp., Armonk, NY, USA).

This study was approved by the institutional Research Ethics Committee under protocol numbers 128/11, CAAE 0104.0.007.000-11, on October 21 2011.

## Results

Of the 703 patients identified, 77.4% were men, 56.8% were aged under 60 years (mean ± SD = 59.03 ± 11.89 yr.), 62.0% were white, 67.5 % had less than eight years of education, 79.7% were smokers at diagnosis, 73.9% were drinkers, 73.4% had advanced (clinical stage III or IV) OCSCC, and 74.1% of patients died during follow-up. Three hundred and seven patients (43.7%) underwent surgery, either alone or in combination with other therapies. Of these, 82.7% underwent cervical lymphadenectomy and 89.1% had tumor-free resection margins (Table 1).

**Table 1:**
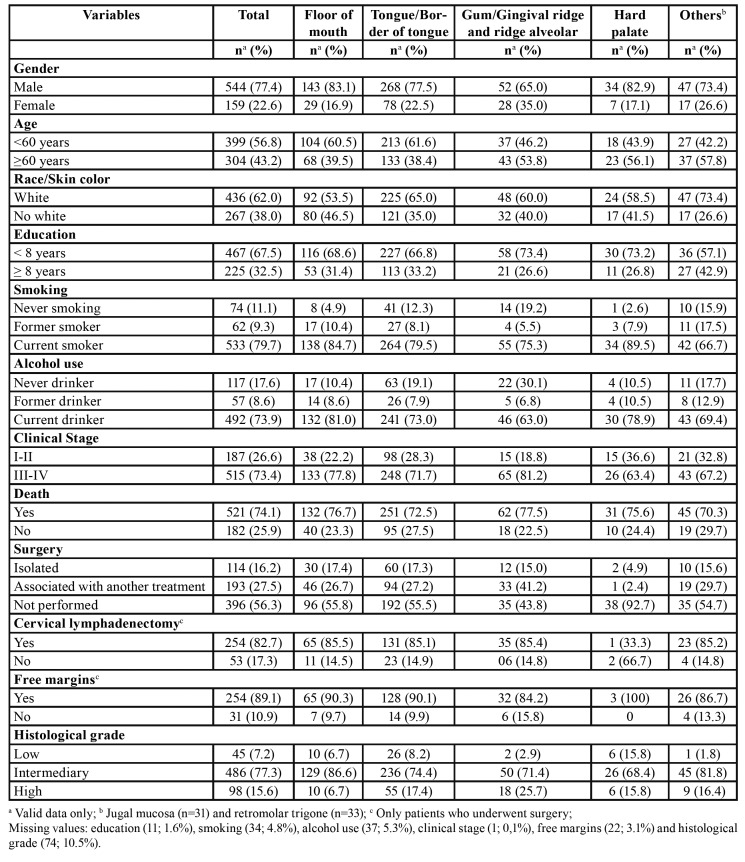
Sociodemographic and clinical characteristics of the study population (n=703).

**Figure 1:**
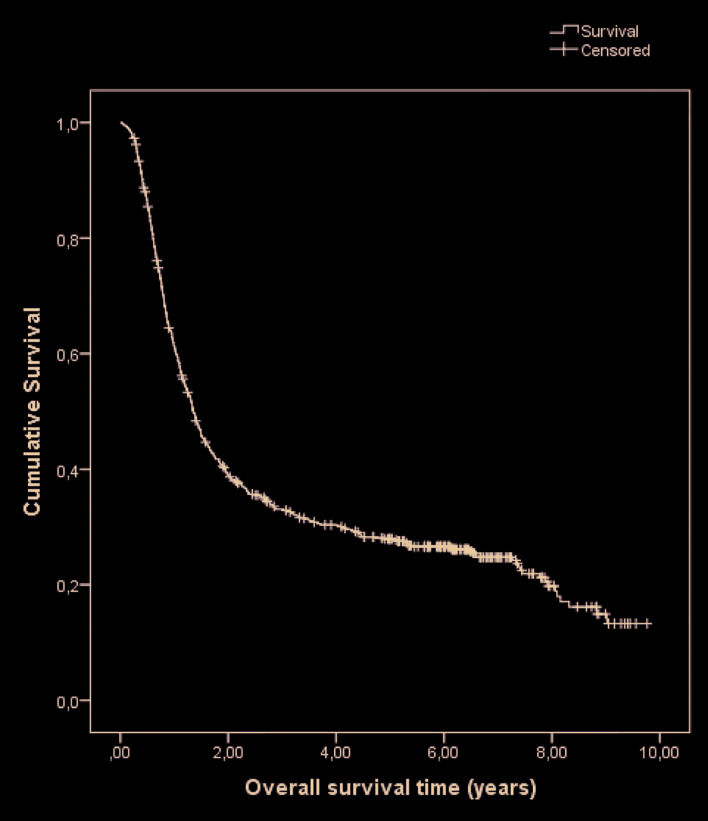
Overall survival of patients with oral cavity squamous cell carcinoma: all patients.

**Figure 2:**
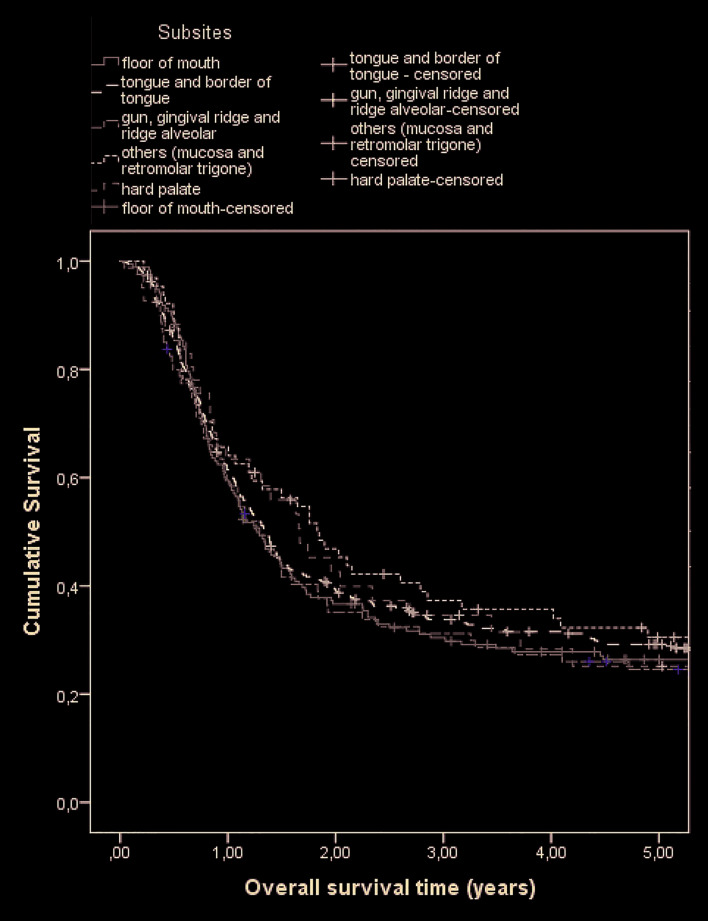
Overall survival of patients with oral cavity squamous cell carcinoma: by tumor subsite.

During the follow-up period, 521 (74.1%) patients died, 43 (8.3%) dying of non-cancer causes. The calculated overall survival of the cohort was 39.1% at two years and 27.9% at five years (Fig. 1 and Fig. 2). The median survival time was estimated to be 1.4 years (95% CI: 1.2‒1.5). Results of overall survival analysis by tumor subsite are presented in Table 2. The variables associated with the risk of death are shown in Table 3.

**Table 2:**
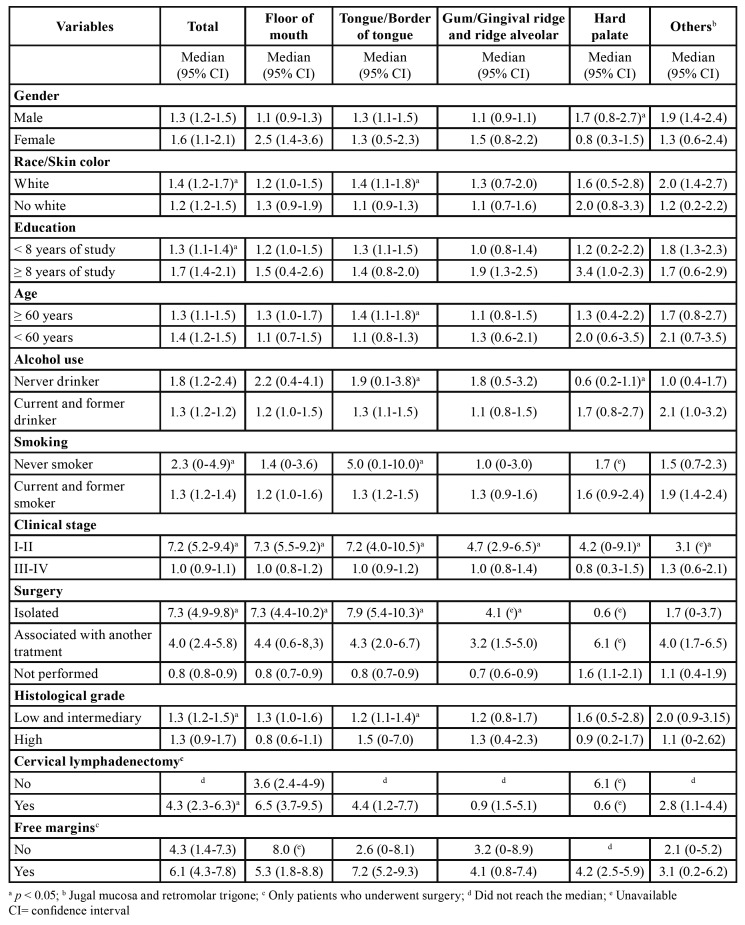
Median survival (years) for patients diagnosed with SCC (n=703).

**Table 3:**
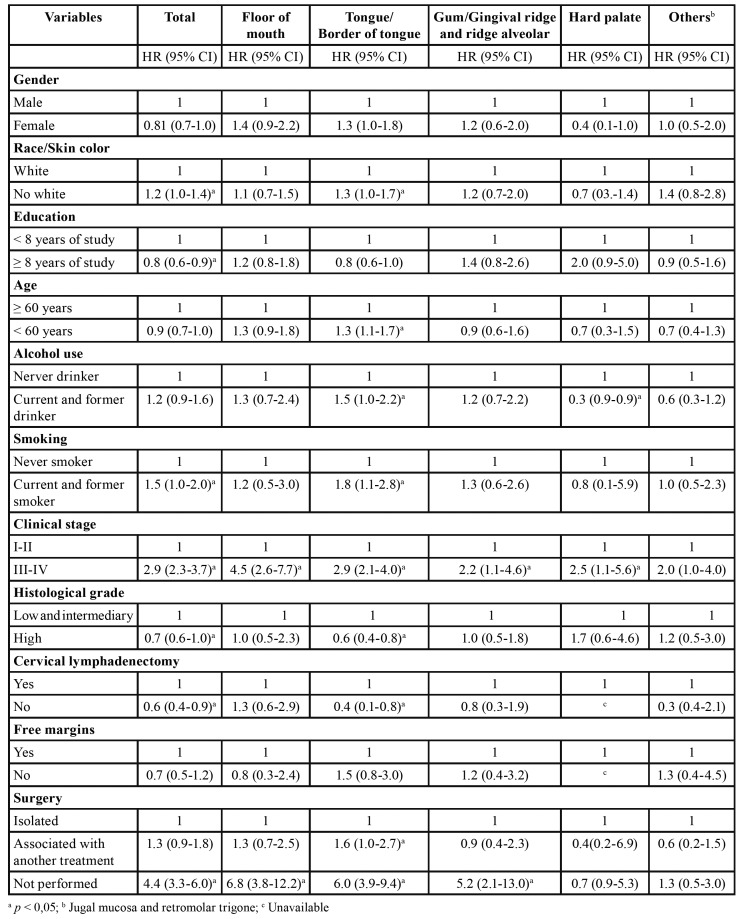
Factors associated with overall survival in patients with SCC (n=703).

The variables independently associated with OCSCC death by tumor subsite are presented in Table 4.

**Table 4:**
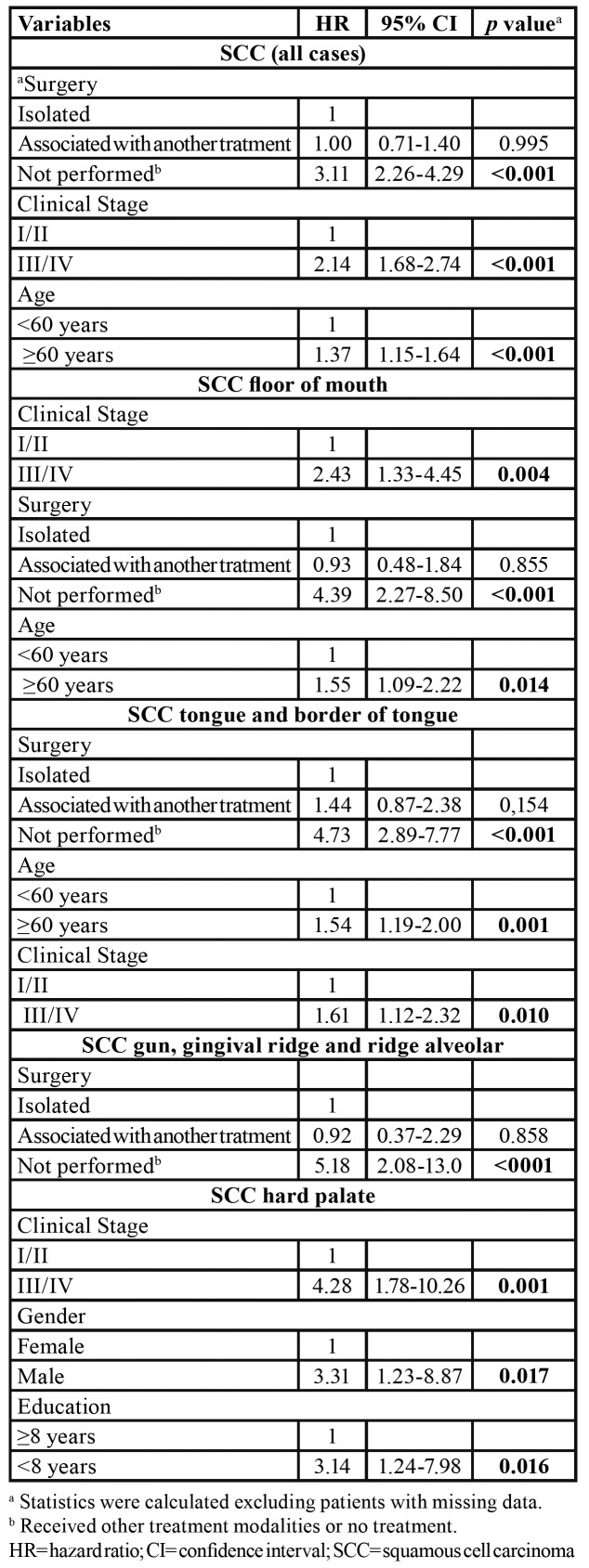
Independent prognostic factors for death of OCSCC patients.

For the overall cohort, nonoperative treatment (HR 3.11; 95%CI 2.26-4.29; *p*<0.001), advanced stage (HR 2.14; 95%CI 1.68-2.74; *p*<0.001), and age > 60 years at diagnosis (HR 1.37; 95%CI 1.15-1.64; *p*<0.001) were independently associated with the risk of death. Advanced stage (HR 2.43; 95%CI 1.33-4.45; *p*= 0.004), nonoperative treatment (HR 4.39; 95%CI 2.27-8.50; *p*<0.001), and age > 60 years at diagnosis (HR 1.55; 95%CI 1.09-2.22; *p*= 0.014) were independently associated with the risk of death for floor of mouth tumors, whereas nonoperative treatment (HR 4.73; 95%CI 2.89-7.77; *p*<0001), age > 60 years (HR 1.53; 95%CI 1.19-2.00; *p*= 0.001) and advanced stage (HR 1.61; 95%CI 1.12-2.32; *p*= 0.010) were independently associated with the risk of death for tongue and border of tongue tumors. In addition, nonoperative treatment (HR 5.18; 95%CI 2.08-13.0; *p*<0001) was independently associated with the risk of death for gum and gingival ridge tumors. For hard palate tumors, advanced stage (HR 4.28; 95%CI 1.78-10.26; *p*= 0.001), male gender (HR 3.31; 95%CI 1.23-8.87; *p*= 0.017), and < eight years of education (HR 3.14; 95%CI 1.24-7.98; *p*= 0.016) were independently associated with the risk of death. Lastly, multivariate analysis was not performed for jugal mucosa and retromolar trigone because no variables were significant on the univariate analysis.

## Discussion

In this study, 703 patients with OCSCC who were treated at a single cancer center in Brazil were retrospectively identified. Most patients were men with low level of education who smoked and drank. The most prevalent tumor site was the tongue (49.2%). More than 2/3 of patients were diagnosed at an advanced stage and died during follow-up. Nonoperative treatment, advanced stage, and age > 60 years at diagnosis were independently associated with the risk of death. However, these factors varied by tumor subsite.

Surgery remains the most important curative treatment modality that impacts on the prognosis of oral neoplasms. The reasons why patients are not submitted to surgery may be related to primary radiotherapy treatment, comorbidities that would prevent surgery, the presentation of unresecTable/incurable (advanced staging) disease or patient preference (8). The prognosis for early-stage disease is relatively good, but 40 to 60% of patients have advanced-stage disease at diagnosis (10). If cancer is diagnosed and treated early, the risk of micrometastatic spread of disease and treatment-related morbidity decreases. In addition, patients with advanced-stage oral cancer have significantly worse health-related quality-of-life scores compared to patients treated for early-stage disease (11). Elderly people are significantly less likely to undergo oral cavity resection despite the increased incidence in this group. The reasons for this may be related to the presence of comorbidities that would contraindicate them from surgery, treatment refusal or being treated in centres that are less likely to recommend surgery due to age, leading to an undertreated elderly population (12).

Gourin and Podolsky, in 2006, identified 1,128 patients diagnosed with head and neck SCC from 1985 to 2002 in Georgia, USA and showed that black race/skin color, advanced TNM stage, 2‒3 comorbidities, inoperable disease, and tumor subsite were associated with poorer overall survival (13). In the current study, stage III and IV disease (SCC of the floor of mouth, tongue and border of tongue, hard palate and nonoperative treatment (SCC of the floor of mouth, tongue and border of tongue, gum, gingival ridge, and alveolar ridge mucosa) were associated with poor survival.

In a study that investigated the effect of race and gender on long-term survival of oral and oropharyngeal cancer in 22,162 patients identified in the US Surveillance, Epidemiology and End Results (SEER) database from 1975 to 1986, Osazuwa-Peters *et al*. reported that black race/skin color, male gender, low socioeconomic status, living without a partner, advanced stage disease at diagnosis, and nonoperative treatment were associated with the poorest overall survival rates; in their study, tumor subsite was also associated with survival (14). Similarly, in the current study, men (SCC of the hard palate), patients with advanced stage disease (SCC of the floor of mouth, tongue and border of tongue, hard palate), and those who received nonsurgical treatment (SCC of the floor of mouth, tongue and border of tongue, gum, gingival ridge, and alveolar ridge mucosa) had poorer survival. In contrast, race/skin color did not influence survival.

Van Dijk *et al*., in 2016, identified 13,108 patients diagnosed with OCSCC in The Netherlands from 1991 to 2010 and found that male gender, older age, advanced stage disease, nonoperative treatment, and late treatment were significantly associated with poor five-year overall survival in OCSCC patients. In addition, overall survival varied by tumor subsite (8). Similarly, in the current study, male gender (SCC of the hard palate), older age (SCC of the floor of mouth, tongue and border of tongue), advanced stage disease (SCC of the floor of mouth, tongue and border of tongue, hard palate), and nonoperative treatment (SCC of the floor of mouth and gum, tongue and border of tongue, gingival ridge, and alveolar ridge mucosa) were associated with poor survival.

In a retrospective study of 2,738 patients who received treatment between 1990 and 2011 at seven international cancer centers, Amit *et al*. reported that surgery after 2000, negative tumor margins, adjuvant therapy, and early stage disease were independent predictors of better five-year overall survival (7). Similarly, in our study advanced stage disease was associated with poor prognosis in cases of SCC of the floor of mouth, tongue and border of tongue, and hard palate.

In a retrospective study that identified 909 patients with SCC of the oral cavity and pharynx treated at the M. D. Anderson Cancer Center from 1984 to 1993, Moore *et al*. reported that patients 60 years of age and older, African-Americans, and those who received nonsurgical treatment had a higher risk of dying (6). Similar results were observed in the current study for patients with SCC of the floor of mouth (age and treatment modality), tongue and border of tongue (age and treatment modality), and gum, gingival ridge and alveolar ridge mucosa (treatment modality).

To our knowledge, only two studies have examined the independent prognostic factors for survival in specific subsites of the oral cavity (15,16). In a study that described the incidence and determinants of survival of 1,489 patients with SCC of the hard palate between 1973 and 2014 using the SEER database, Alonso *et al*. found that advanced age, nonoperative treatment, radiation therapy, tumor stage and grade were independently associated with worse overall survival (15). In the current study, in addition to tumor stage, male gender and low education level were also independently associated with poor survival in patients with SCC of the hard palate.

In a study that examined the survival of 62 patients with tongue cancer who received treatment between 2009 and 2012 in Indonesia, Sutandyo *et al*. reported that stage T3/T4 tumors were associated with a significantly increased risk of death compared to T1/T2 tumors. However, in their study, stage was not significantly associated with overall survival (16). Contrarily, stage III and IV disease was associated with an increased risk of death in our study, even though independent factors associated with the risk of death identified in our sample such as age ≥ 60 years was not investigated by Sutandyo *et al*. (16).

Limitations of this study include its retrospective secondary nature and the number of patients with missing data for some variables, which was ameliorated by the search of physical and electronic medical records. However, the authors assumed that the small percentage of missing data (< 5%, except for histological grade) did not bias the results. Moreover, prognostic factors for OCSCC including surgical (reconstructive technique, tracheostomy) and clinical (body mass index, albumin and hematocrit levels, Human Papilloma Virus [HPV] and Epstein-Barr virus [EBV] infections) parameters, tumor histology (histologic type, perineural invasion, lymphovascular invasion, lymph node positivity), and tumor markers (Ki67, epidermal growth factor receptor [EGFR], interleukin-2) were not examined. In addition, the small sample size for the Gum/Gingival ridge and alveolar ridge (n=74), Hard palate (n=41) and Others (n=64) may not have sufficient power to detect differences between categories as being statistically significant (type II error). Nevertheless, a strength of this study is that it is the first to explicitly examine the prognostic factors associated with overall survival in OCSCC stratified by tumor subsite in a Brazilian population from a single cancer center.

## Conclusions

In this study, different independent prognostic factors were associated with the risk of OCSCC death depending on tumor subsite. Tumor stage was not an independent predictor of death only in SCC of the gum, gingival ridge, and alveolar ridge. Surgical treatment was an independent predictor of death in SCC of the floor of mouth, tongue, and gum. It should be noted that tumor staging at diagnosis is essential in deciding appropriate treatment. In addition, age > 60 years was associated with risk of death in SCC of the floor of mouth and tongue/border of tongue. In the latter tumors, race/skin color other than white was also associated with poor survival. Finally, male gender and < eight years of education were independent predictors of death in SCC of the hard palate.
